# Brain areas involved in the acupuncture treatment of AD model rats: a PET study

**DOI:** 10.1186/1472-6882-14-178

**Published:** 2014-05-31

**Authors:** Yangjia Lu, Yong Huang, Chunzhi Tang, Baoci Shan, Shaoyang Cui, Junjun Yang, Junqi Chen, Renyong Lin, Huiling Xiao, Shanshan Qu, Xinsheng Lai

**Affiliations:** 1 School of Traditional Chinese Medicine, Southern Medical University, Guangzhou 510515, China; 2 Clinical Medical College of Acupuncture, Moxibustion and Rehabilitation, Guangzhou University of Chinese Medicine, Guangzhou, China; 3 Key Laboratory of Nuclear Analytical Techniques, Institute of High Energy Physics, Chinese Academy of Sciences, Beijing 100049, China; 4 Traditional Chinese Medicine Department of the Second Clinical School, Guangdong Medical College, Donguan, Guangdong Province, China; 5 The TCM Hospital of Futian District of Shenzhen, Shenzhen, China

**Keywords:** PET, Needling, ST36, Rat, AD

## Abstract

**Background:**

Acupuncture may effectively treat certain symptoms of Alzheimer’s disease (AD). Although several studies have used functional brain imaging to investigate the mechanisms of acupuncture treatment on AD, these mechanisms are still poorly understood. We therefore further explored the mechanism by which needling at ST36 may have a therapeutic effect in a rat AD model.

**Methods:**

A total of 80 healthy Wistar rats were divided into healthy control (n = 15) and pre-model (n = 65) groups. After inducing AD-like disease, a total of 45 AD model rats were randomly divided into three groups: the model group (n = 15), the sham-point group (n = 15), and the ST36 group (n = 15). The above three groups underwent PET scanning. PET images were processed with SPM2.

**Results:**

The brain areas that were activated in the sham-point group relative to the model group were primarily centred on the bilateral limbic system, the right frontal lobe, and the striatum, whereas the activated areas in the ST36 group were primarily centred on the bilateral limbic system (pyriform cortex), the bilateral temporal lobe (olfactory cortex), the right amygdala and the right hippocampus. Compared with the sham-point group, the ST36 group showed greater activation in the bilateral amygdalae and the left temporal lobe.

**Conclusion:**

We concluded that needling at a sham point or ST36 can increase blood perfusion and glycol metabolism in certain brain areas, and thus may have a positive influence on the cognition of AD patients.

## Background

Alzheimer’s disease (AD) is the most common form of dementia in elderly individuals and is associated with progressive memory loss and cognitive dysfunction. AD is characterised by extensive cortical neuropathology, including extracellular amyloid-containing neural plaques, intracellular neurofibrillary tangles, and cell and synapse loss in the cortical and subcortical regions of the human brain [1-4]. Research is being performed around the world to find a cure for this disease.

Acupuncture has therapeutic effects on Alzheimer’s disease [5-10]. Many studies provide different explanations as to how acupuncture may cure Alzheimer’s disease [11,12]. However, the mechanism is still unknown. This study was based on functional brain imaging using positron emission tomography (PET) to explore the mechanism of the therapeutic effect of acupuncture on AD. PET is a non-invasive imaging technique and can reveal the level of cellular metabolism. This technique has been used to explore acupoint specificity and the relationship between needling specificity and the response in cerebral regions [13]. Jia et al. [14], Dong et al. [15], and Yin et al. [16,17] have all performed similar studies based on PET to determine how needling works. Their results showed that needling at different acupoints can stimulate different brain regions. Our literature review revealed the following: (1) All of these studies were performed on humans, and there have been no PET studies to determine the effects of needling in rats. (2) No functional brain imaging studies have been performed on rat models of AD. This study is the first to study the needling of rats using PET. We selected ST-36 (Zhusanli) as the needling point with the aim of determining how needling at ST-36 affects the brain.

## Methods

### Design

A randomised, controlled animal experiment.

### Time and setting

The study was performed at the Experimental Animals Center of the China Academy of Chinese Medical Sciences, the PET-CT centre of the Experimental Animals Center of the General Hospital of PLA, and the Institute of High Energy Physics of the Chinese Academy of Sciences from January 2011 to October 2012.

### Ethical statement

Animal care and sacrifice were conducted according to methods approved by the Animal Care and Use Committee, Guangzhou University of Traditional Chinese Medicine (TCM), Guangzhou, China. All experiments were performed in accordance with the National Institute of Health Guide for the Care and Use of Laboratory Animals. This experiment was approved by the Ethics Committee of Guangzhou University of TCM (SPF20110032).

### Materials

#### Reagents and instruments

Reagents and instruments were used in this research were showed as Table 1.

**Table 1:** Reagents and instruments used in this study

**Reagents and instruments**	**Type**	**Source**
D-Galactose (d-gal)	-	Shanghai No. 2 reagent company
Ibotenic acid (IBA)	-	Sigma Chemical Co. P.O.
sodium pentobarbital	-	Beijing Chemical Reagent Company
sodium benzylpenicillin	-	Harbin Group Pharmaceutical Factory
Normal sodium, NS	-	Guangxi Nanning Bai-hui Pharmaceutical Group Co., LTD
stereotaxic apparatus	WDT-2	Xian Northwest Photoelectric Instrument Factory
^18^ F-FDG	-	General Hospital of PLA
MicroPET imaging system	nanoScan PET	SIEMENS, Germany
ECAT EXACT HR + PET imaging system	HR+	SIEMENS, Germany
Animal breathing anaesthesia machine	VME	Matrx Company, America
isoflurane	-	Hebei Jiu-pai Pharmaceutical Co., LTD
Rat Y-maze	RD1102-YM-R	Zhenhua Teaching Instrument Yuanyang, Hebei

#### Experimental animals

A total of 80 healthy 8-week-old Wistar rats (40 males and 40 females) weighing 200–250 g were provided by the animal centre of the China Academy of Chinese Medical Sciences. The entire experimental procedure was in accordance with the Guidelines for the Care and Use of Laboratory Animals of the Ministry of Science and Technology of the People’s Republic of China.

### Methods

#### Primary experimental groups

The 80 rats were bought from the animal laboratory of Guangzhou University of TCM, Guangzhou, and housed in the Experimental Animals Center of the China Academy of Chinese Medical Sciences. All rats were housed in separate cages under conditions of controlled illumination (12:12 h light/dark cycle), humidity, and temperature (18–22°C). After 1 week of acclimatisation to the home cage, the rats were randomly assigned to the healthy control (n = 15, 8 males and 7 females) or pre-model (n = 65, 32 males and 33 females) groups.

#### Generation of the animal model

The healthy control (HC) group was not subjected to any treatment. The pre-model group received D-gal (20 mg in 2 ml 0.9% saline, intraperitoneal injection every day) for 6 weeks. After 6 weeks of intraperitoneal D-gal injections, the pre-model rats were anesthetised with pentobarbital (50 mg/kg). The head of each rat was placed in a stereotaxic frame, and a needle with a syringe (Hamilton) was placed in the right NBM, 0.9 mm posterior and 2.8 mm lateral to the bregma and 6.9 mm vertically from the skull surface. A stereotaxic injection of 6 Ag of ibotenic acid in 0.3 Al of phosphate-buffered saline (pH 7.4) was performed at a rate of 0.1 Al/min. The syringe was left in place for more than 3 min at the site of injection. The position of the lesion was confirmed using Nissl staining. Two weeks after the IBO injection [18-20], all of the animals (including the HC and pre-model animals) were subjected to the Y-maze test. The rats in the pre-model group that met the standards of the AD model were included in the next step. A total of 45 rats (23 males and 22 females) that fulfilled the criteria of the AD model (10 rats, 4 males and 6 females, died during the D-gal treatment, and 5 rats, 3 males and 2 females, did not meet the criteria for the AD model) and 15 healthy rats were included.

#### Secondary experimental groups

The 45 AD model rats were divided into three groups using a random number table: the model group (n = 15, 7 males and 8 females), the sham-point group (n = 15, 7 males and 8 females), and the ST36 group (n = 15, 8 males and 7 females). The three groups and the HC group underwent PET scanning.

#### ST36 and sham point location

**Figure 1:**
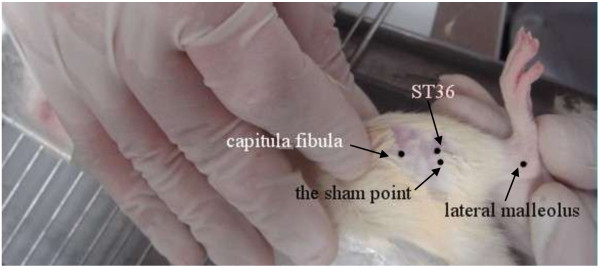
Location of ST36 and the sham point.

The ST36 point is located 5 mm directly below the capitulum fibulae, and the sham-point used was located 2 mm away from ST36 (Figure 1).

#### Needling method

Needling was performed on rats in the sham-point group and the ST36 group the day after the AD model rats were assigned to groups. Needling was performed at the Experimental Animals Center of the China Academy of Chinese Medical Sciences, from 08:00 to 12:00. Each rat was needled one time per day, and each single needling lasted for almost 30 min. During needling, rats were fixed only by the hand of the operator; furthermore, they were penetrated bilaterally. Needling was performed as follows: after the overlying skin was sterilised with an iodine tincture and alcohol, sterile acupuncture needles (0.10 mm in diameter and 0.9 mm in length, manufactured by the Shuzhou Medical Appliance Factory, Shuzhou, China) were inserted perpendicularly into the selected points to a depth of 0.5–0.6 mm. The needles were gently twisted, lifted and reinserted for even reinforcing and reducing. The twisting was performed within the range of 90–180° at a rate of 60–90 times/min. The lifting and thrusting was performed within the range of approximately 0.1–0.2 mm at a rate of 60–90 times/min.

#### [^18^ F] fludeoxyglucose (FDG)-PET imaging

All rats were sent to the PET-CT centre of the Experimental Animals Center of the General Hospital of PLA at 8:00 am after 24 h of fasting and were then subjected to the following procedure: (1) The blood sugar level was determined. (2) The rats were allowed to rest for 20 min in a dark room. (3) A tracer (^18^ F-FDG, synthesised with Mini Tracer accelerator, 0.11 mci/kg dosage) was injected via the tail vein. (4) The rats were allowed to rest for 40 min. After tracer injection, the HC group and the model group rats were allowed to move freely in a small box for 30 min. Then, 30 min later, the HC group and the model group rats received gas (isoflurane) anaesthesia; the total time of gas anaesthesia was almost 10 min. The sham-point group rats and the ST36 group rats underwent needling 2 min before the [^18^ F]FDG injection. [^18^ F]FDG was injected via the tail vein with the needle still in the acupoint (or sham-point). After finishing the injection (which was performed over almost 1 min), the manipulation of the needle was continued as before for an additional 7 min. The total needling time was approximately 10 min. Then, the rats were allowed to move freely in a small box, as for the model group. Twenty minutes later, the rats were placed in a gas anaesthesia box for anaesthesia, and 10 min later, when the rats were fully anaesthetised, they underwent PET scanning. (5) The rats were subjected to a PET scan. PET scans were performed on a Biograph Duo BGO scanner (Siemens, Germany). The images covered the whole brain and were parallel to the AC-PC line. Image acquisition was started after a 40 min uptake period (bed: 1; collection mode: 3D; slice thickness: 3 mm; slice interval: 1.5 mm; matrix size: 256 × 256; total counts: 3 × 109). Upon the completion of data acquisition, the images were reconstructed using ordered subset expectation maximisation (OSEM) with 6 iterations and 16 subsets.

#### Statistical analysis

The preprocessing and data analysis were performed using our self-developed toolbox for voxel-wise analysis of rat brain images based on SPM8 (Welcome Department of Cognitive Neurology, London, UK), which comprised a FDG-PET rat brain template and atlas in Paxinos & Watson space [21]. The images of all rats in the model group, the sham-point group, the ST36 group and the healthy controls were preprocessed using the following main steps. (1) Segmentation. All the FDG-PET functional images were registered to the average rat brain with extracranial tissues using intensity-based twelve-parameter affine transformation. The intracranial mask image was employed to automatically crop brain tissues from non-brain tissues in all images. (2) Normalisation. Each intracranial individual image was spatially normalised with the intracranial FDG-PET rat brain template in SPM8. (3) Smoothing. The spatially normalised functional images were smoothed by a Gaussian kernel of 2*2*4 (after zooming) full width at half-maximum (FWHM). Preprocessed images were analysed within SPM8 based on the framework of the general linear model. To identify the difference of FDG signals between the rats with AD and the healthy controls, two-sample t-test was performed using SPM8. Proportional scaling was applied to account for global confounds. Brain regions with significant FDG changes in rats with AD were yielded based on a voxel-level height threshold of p < 0.001 (uncorrected) and a cluster-extent threshold of 50 voxels.

## Results

**Figure 2:**
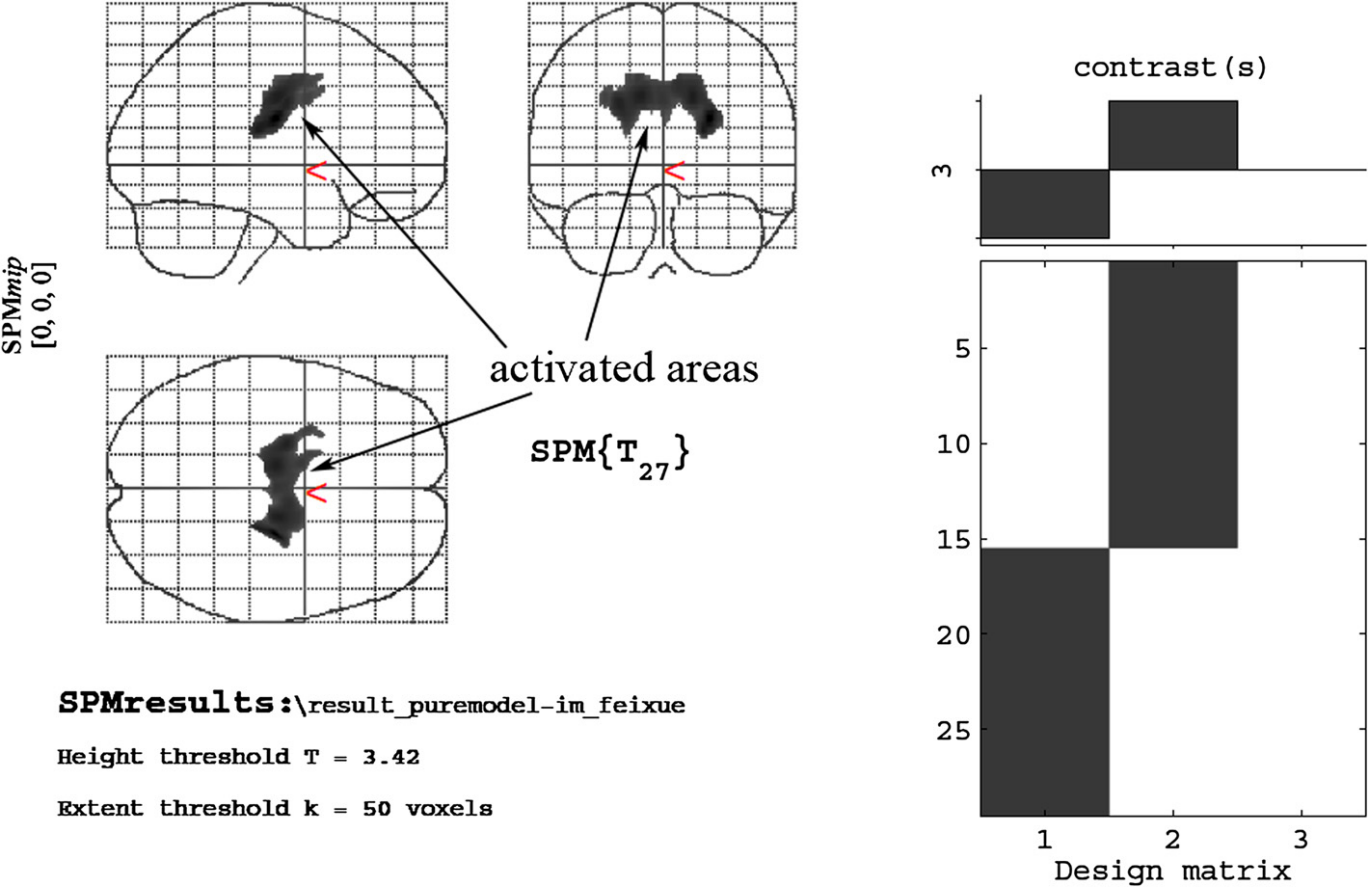
Cerebral areas activated in the sham-point group vs. the model group.

**Figure 3:**
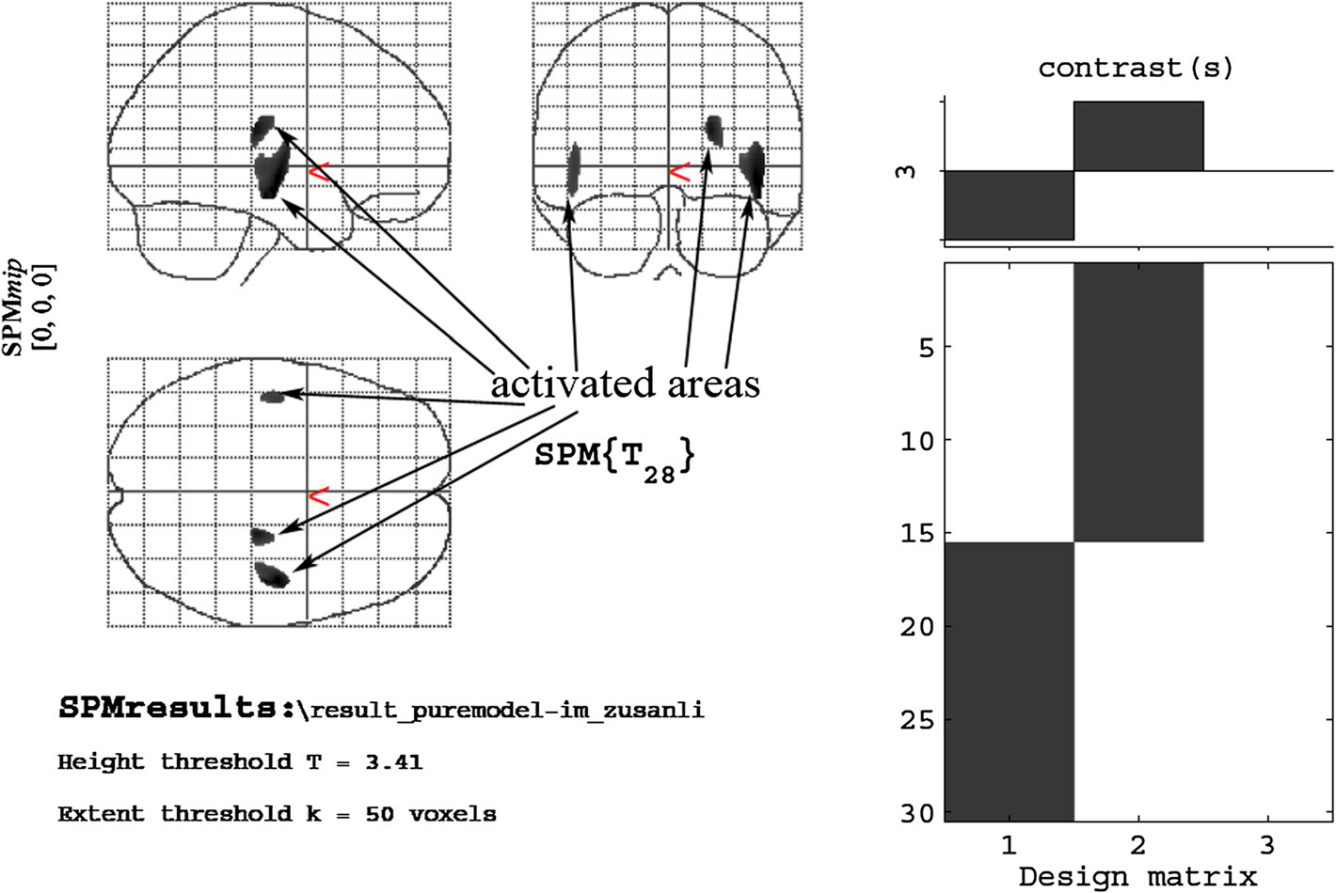
Cerebral areas activated in the ST36 group vs. the model group.

**Figure 4:**
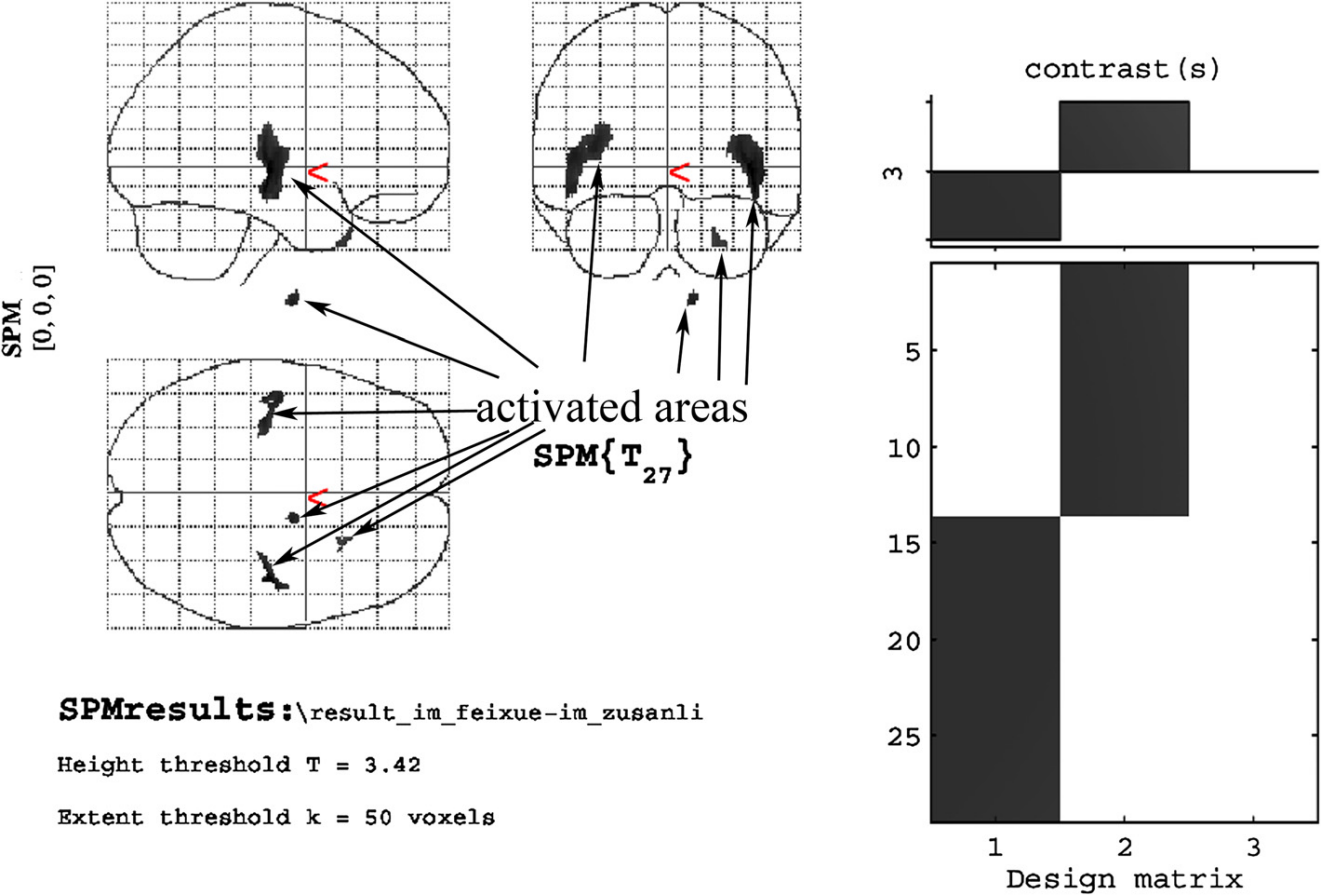
Cerebral areas activated by needling ST36 vs. the sham point.

Compared with the model group, the sham-point group exhibited a higher level of glycol metabolism in the right nucleus accumbens, the septal area of the right limbic system, the orbital cortex of the right frontal lobe, the left corpus callosum, the left nucleus accumbens, and the left striatum (Table 2, Figure 2). Compared with the model group, the ST36 group exhibited a higher level of glycol metabolism in the pyriform cortex of the right limbic system, the olfactory cortex of the right temporal lobe, the right amygdaloid body, the right hippocampus, the pyriform cortex of the left limbic system, and the olfactory cortex of the left temporal lobe (Table 3, Figure 3). Compared with the sham-point group, the ST36 group showed a higher level of glycol metabolism in the right amygdaloid body, the olfactory cortex of the left temporal lobe, and the left amygdaloid body (Table 4, Figure 4).

**Table 2:** Sham-point group compared with the model group

**Anatomical structure**	**Volume**	**T-Average**	**x**	**y**	**z**
R Limbic System: Septal Area	173	3.853346	41	16	62
R Frontal Lobe: Orbital Cortex	114	3.771888	44	36	78
L Corpus Callosum	137	3.714816	73	39	78
L Nucleus Accumbens	699	4.278525	71	26	73
L Limbic System: Septal Area	140	3.916688	61	26	72
L Striatum	1395	3.990151	82	32	71

**Table 3:** ST36 group compared with the model group

**Anatomical structure**	**Volume**	**T-Average**	**x**	**y**	**z**
R Limbic System: Piriform Cortex	115	3.799693	14	26	46
R Temporal Lobe: Olfactory Cortex	172	4.328225	14	24	41
R Amygdaloid Body	364	3.790134	16	28	50
R Hippocampus	119	4.208104	16	26	45
L Limbic System: Piriform Cortex	120	3.587087	107	25	49
L Temporal Lobe: Olfactory Cortex	115	3.606412	107	27	52

**Table 4:** ST36 group compared with the sham-point group

**Anatomical structure**	**Volume**	**T-Average**	**x**	**y**	**z**
R Amygdaloid Body	170	4.421505	19	22	50
L Temporal Lobe: Olfactory Cortex	117	4.256156	107	27	54
L Amygdaloid Body	128	4.125938	96	19	56

## Discussion

It is true that studies on acupoints should be performed in humans, but we believe that studies conducted with animals also have irreplaceable significance. To date, several studies of acupuncture using functional brain imaging have been performed. However, all we know from the past studies is that needling at certain acupoints can stimulate certain brain regions. Additionally, how these activated brain regions affect the target organs and cure diseases remains unknown. Many researchers have thought about this gap in knowledge and have appealed to others to perform studies in this field [22,23]. However, these studies may have negative consequences on human subjects, and may require the analysis of brain tissue or another organ that cannot obtained from live humans. In contrast, it is easy to take these tissues from rats. Therefore, it may be best to perform preliminary studies in rats.

ST36 is the primary point of the four main acupoints used in traditional medical practice that has distinctive accommodation and therapeutic effects. This point is associated with more than ten common diseases, including digestive, respiratory, circulatory, urinary and nervous disorders. ST36 has been used for a long time in TCM as the main point to treat AD. In addition, many recent studies have demonstrated that ST36 plays an important role in the treatment of AD [24,25]. Based on these factors, we chose ST36 as the research point.

The results of this study can be grouped into three parts: (1) the brain regions with higher glycol metabolism in the sham-point group relative to the model group, (2) the brain regions with higher glycol metabolism in the ST36 group relative to the model group, and (3) the brain regions with higher glycol metabolism in the ST36 group relative to the sham-point group.

Many functional brain-imaging studies have investigated the effect of needling at ST36. Yin l et al. used PET to perform functional brain imaging while needling the ST36 points of six healthy volunteers, and they found that needling at ST36 can increase glycol metabolism in the hypothalamus, the head of the caudate nucleus, the temporal lobe, the left cerebellum, the postcentral gyrus, and the brain stem [17]. Wu Zhiyuan et al. used the BOLD sequence to observe the changes in the brain when performing acupuncture at the right ST36 of 12 healthy volunteers. The data were analysed and compared using SPM2, and this analysis revealed that there were increases in brain activity in the left parahippocampal gyrus, the left middle temporal gyrus, the left superior temporal gyrus, the right superior temporal gyrus and the right supramarginal gyrus when acupuncturing ST36 [26]. A study by Zhou investigating the effect of needling at ST36 in AD showed very similar result to our study. The differences between the two studies were as follows: (1) Zhou used fMRI, but we used PET; and (2) Zhou studied humans, but we studied rats. Zhou found that the activated regions were primarily the inferior frontal gyrus, the middle frontal gyrus, the superior frontal gyrus, the transverse temporal gyrus, the left cerebrum, the left superior frontal gyrus, the middle frontal gyrus, the precentral gyrus, the hippocampal gyrus, the cingulate gyrus, the postcentral gyrus, and the paracentral lobule of the right cerebrum. Zhou concluded that exciting ST36 could improve the cognitive ability of AD patients and that the mechanism may involve the activation of the cognition-related regions of the frontal and temporal lobes and the marginal system, as well as the cognition-related region of the cerebellum [27]. Other studies have shown a higher level of glycol metabolism in the No. 1 and 2 somesthetic areas, the No. 1 and 2 body movement areas, the premotor area, the submotor area, the inferior parietal lobe, the insula lobe, the cingulate gyrus, the prefrontal lobe, the temporal lobe, the hippocampus, the head of the caudate nucleus, the lenticular nucleus, the putamen, the nucleus accumbens, the thalamus, the hypothalamus, the paraventricular nucleus, the pons Varolii and the cerebellum [28-31]. Our results were quite different from most past results but were similar to Zhou’s results, which may be because Zhou’s study and our study included needling at ST36 in AD instead of healthy controls.

Senile plaques, neurofibrillary tangles and neuron loss are the three main neuropathologic changes in AD patients. The senile plaques and neurofibrillary tangles first occur in the medial temporal lobe (MTL), which includes the entorhinal cortex, the hippocampus, the parahippocampal gyrus and the amygdala. When AD progress, these three main features can spread to the neopallium, including the frontal lobe and the parietal lobe, and can eventually reach the sensory cortex [32]. In recent years, researchers have carried out many studies on the central pathological mechanism of AD. It has been generally acknowledged that in every pathologic stage of AD, the damaged spreads to different brain regions. These damaged brain regions have lower blood perfusion than is normal for elderly individuals. A previous study provided evidence that the deficient blood perfusion in damaged brain regions may be one of the main causes of the progressive cognitive deficit in AD patients [33].

Our results showed that the brain areas activated in the sham-point group relative to the model group were mainly centred on the bilateral limbic system, the right frontal lobe, and the striatum, whereas the activated areas in the ST36 group were mainly centred on the bilateral limbic system (pyriform cortex), the bilateral temporal lobe (olfactory cortex), the right amygdala and the right hippocampus. Compared with the sham-point group, the ST36 group showed activated brain regions in the bilateral amygdalae and the left temporal lobe.

The limbic system contains structures that are involved in emotion, memory and sensation. It is generally acknowledged that the limbic lobe of the limbic system is primarily located in the left hemisphere cortex. Our results revealed an apparent activation of the limbic system when needling the sham-point and ST36. Therefore, needling at these two points may improve memory and cognitive function, but these results may also be due to the rats’ sensations, such as pain and fear.

This study had several limitations: (1) Our AD model was based on the intraperitoneal injection of D-gal combined with the injection of IBO into the basal nuclei. This type of AD model rat mimics certain symptoms of AD but does not completely recapitulate human AD. We based our AD model on several previous studies [18-20]. (2) We tested the development of AD in rats using only a Y-maze test, and analysing some a biochemical indicator may be better.

## Conclusions

According to this study, We concluded that needling at a sham point or ST36 can increase blood perfusion and glycol metabolism in certain brain areas, and thus may have a positive influence on the cognition of AD patients. And this may also give some helps to the clinical doctors.

## Competing interests

All authors declare that they have no financial relationships with biotechnolog manufacturers, pharmaceutical companies, or other commercial entities with an interest in the subject matter or materials discussed in the manuscript.

## Authors’ contributions

YH, CZT and XL conceived and coordinated the study. YH and YL participated in the design of the study. YL, SC and JC performed the study. BS performed the data analysis with SPM2. JY, RL, HX and SQ helped record the data. All authors read and approved the final manuscript.

## Pre-publication history

The pre-publication history for this paper can be accessed here:

http://www.biomedcentral.com/1472-6882/14/178/prepub
